# Reversal of vision after bleomycin injection in orbital lymphangioma

**DOI:** 10.3205/oc000196

**Published:** 2022-03-22

**Authors:** Pratheeba Devi Nivean, Nivean Madhivanan

**Affiliations:** 1 M. N. Eye Hospital, Chennai, Tamil Nadu, India

**Keywords:** bleomycin injection, orbital lymphangioma, reversal of vision

## Abstract

Lymphangiomas are benign hemartomatous tumors that can occur in all parts of the body, but most frequently in the neck (75%), as well as in the axilla and inguinal areas. Surgical removal is very difficult, as it is very fragile and tumor dissection is very difficult. Ultrasound-guided bleomycin injection directly into the cyst collapses the cyst and shrinks the tumor. It reduces proptosis, discomfort, and cosmetic blemish. We present this case who had gross defective vision and relative afferent pupillary defect in her right eye for 10 days which tremendously improved after injection.

## Introduction

Lymphangiomas are non-encapsulated branching lesions lined by a single layer of flat endothelium [1]. The underlying delicate fibrous septa often include lymphoid aggregates, macrophages, and haemorrhages. Though there are various treatment options, the non-invasive approach of injecting sclerosants is a safer and more effective alternative to surgery. We present this case, which showed tremendous improvement in vision after cyst aspiration and bleomycin injection even after 10 days. This procedure can be considered as the first-line therapy for patients with acute bleeding in orbital lymphangioma irrespective of the duration of presentation.

## Case description

**Figure 1:**
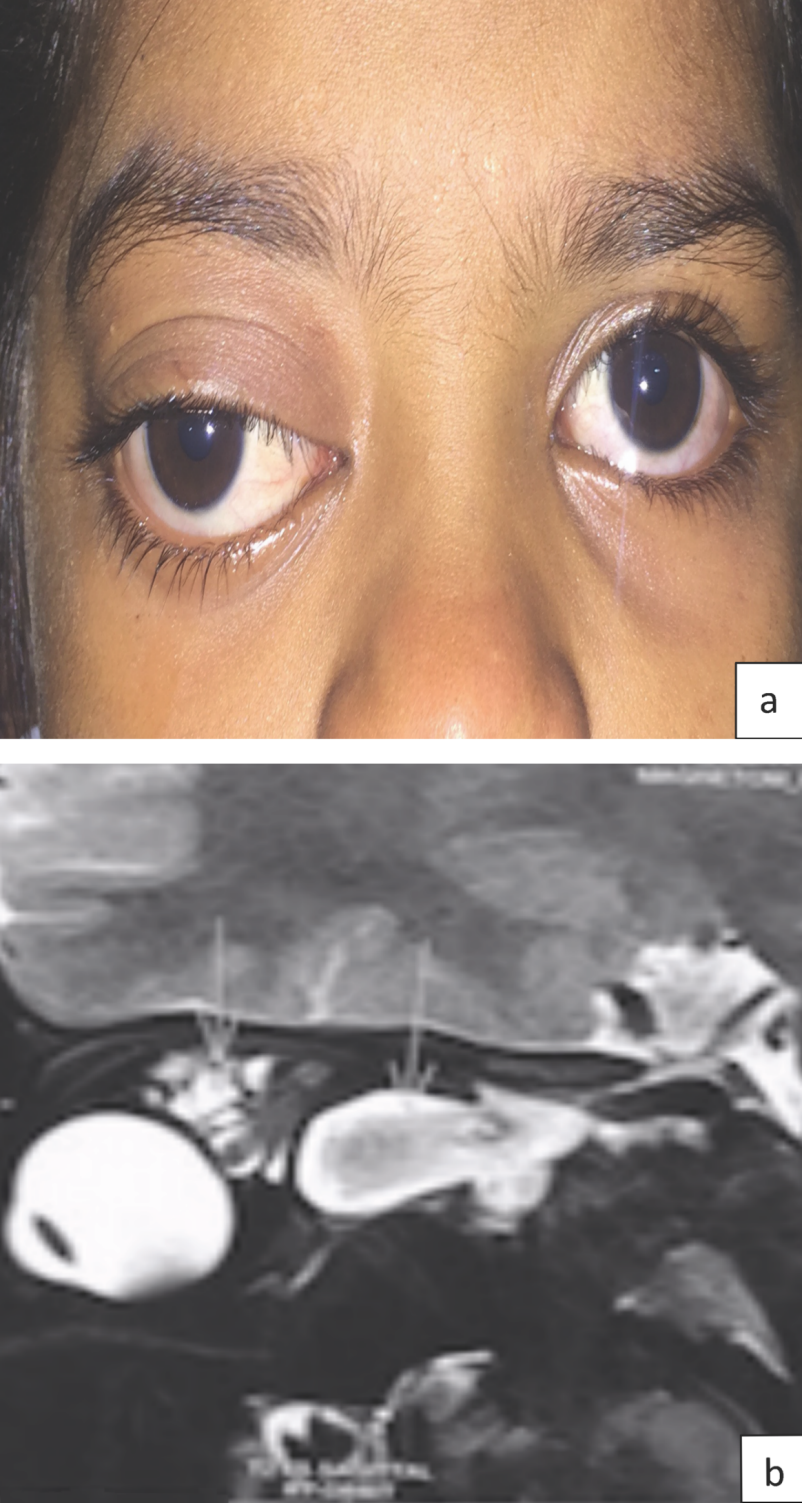
a) Clinical picture of the patient with down and out proptosis; b) MRI orbit sagittal view showing cystic component of lymphangioma above and below the optic nerve

A 6-year-old girl came to us with complaints of acute prominence of her right eye since 10 days. The child has had blurred vision in the right eye since then. The parents gave history of previous prominence of the right eye which aggravated with respiratory tract infection (Figure 1 (Fig. 1)). On examination, the child had a downward and outward proptosis in her right eye. Her vision in the left eye was normal (6/6: n6), and the right eye had hand movement vision. Pupillary examination in the right eye showed relative afferent pupillary defect. The patient’s anterior segment examination was otherwise normal in both eyes. Fundus examination in the left eye was normal, and the right eye retina showed choroidal folds. MRI orbit showed multiple cystic lesions occupying the entire right orbit. Based on the history and MRI findings, we diagnosed the case as lymphangioma with bleeding and optic nerve compromise. Intravenous methylprednisolone 1 g was started immediately and was continued for three days. Meanwhile, cyst aspiration and bleomycin injection was planned in order to reduce the compressive effect on the optic nerve by the cyst.

Under general anaesthesia, the area was cleaned and draped. Lateral canthotomy and cantholysis was done to relieve compression. The cyst was located using an ultrasound B-scan with the help of a retinal surgeon in orbit mode. A 23-gauge needle was passed through the inferior orbital compartment and it was navigated under guidance of the B-scan. The cyst can be clearly visualized as a well-encapsulated lesion with hyperechoic borders and a hypoechoic center. The needle track can be visualized on the screen of the B–scan; and once it punctures a cyst, the fluid is aspirated, then the needle is kept in place, and bleomycin is injected using another syringe. We injected 0.5 IU/kg body weight of bleomycin to the cyst. The ratio of aspirated fluid and bleomycin should be 5:1. Cyst aspiration and sclerosant injection can be done safely.

**Figure 2:**
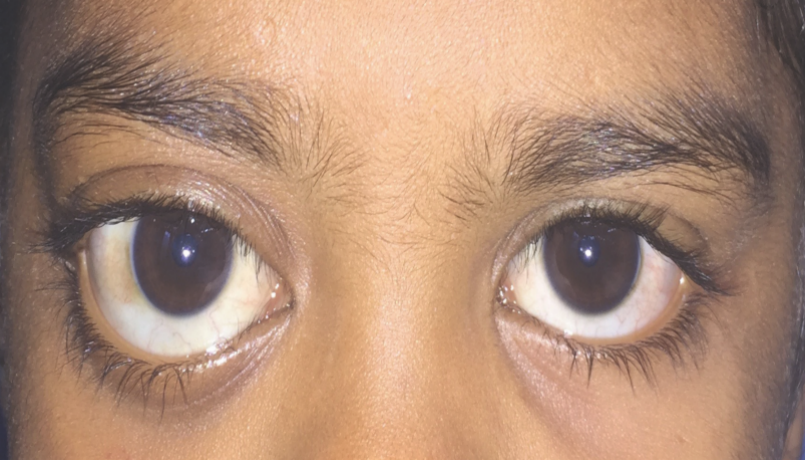
Post-injection photograph of the patient with reduction in proptosis

Post–operatively, the patient was given a course of intravenous steroids followed by oral steroids. The patient responded very well to the first injection. She came back with gross reduction in the proptosis and great improvement in vision (Figure 2 (Fig. 2)). Unfortunately, we do not have a post-procedure MRI as they were not affordable.

## Discussion

Increased orbital pressure can compress the optic nerve and can lead to irreversible vision loss in a few minutes. Rishiem et al. [2] have reported that unless the pressure is released in 90–120 min, there can be irreversible loss of vision. In our case, the patient had compression and hand movement vision for 10 days, and it reversed perfectly after the release of pressure.

Lymphangiomas are low-flow vascular lesions that can be classified based on the location as superficial (subcutaneous/subconjunctival), deep (lesion extending to the orbit), combined (has both superficial and deep components), and complex (involving structures of head and neck). Lymphangiomas can also be classified based on the size as macro and microcyst [3].

Orbital lymphangiomas are ill-defined, non-encapsulated, cystic, and solid orbital lesions that commonly involve the whole orbital spaces including preseptal, postseptal, intraconal, and extraconal compartments. Removal by complete surgical excision is hazardous as it can cause iatrogenic damage to adjacent structures. Moreover, surgical excision can cause disfigurement due to an ugly scar as well. Incomplete excision can lead to recurrence. Since the diagnosis is very characteristic with imaging findings, we did not open the orbit and did not perform histopathology. Hence nonsurgical methods such as intralesional sclerosing agents have been tried as an alternative. Adjunct therapies are safe and effective as well. Non-invasive sclerosant therapy is an efficient alternative for surgery in cases with lymphangioma.

Bleomycin causes a sclerosant effect on the endothelium and collapses the cyst wall [4]. It also induces tumor necrosis factor and causes apoptosis in rapidly dividing cells. Its sclerosing effect on the vascular endothelium was first observed in the treatment of pleural effusions. Bleomycin shrinks the cyst wall and reduces the volume of the cyst. Acute haemorrhage can resolve significantly with time, however the rate and amount of regression is not predictable. It also has the risk of rebleeding.

There is no fixed protocol regarding the number of injections. Raichura et al. [5] gave up to six injections based on the response to the lesion. Nirruddin et al. [6] observed a good response in 3–5 injections. Most of the published literature does not report any recurrence up to 36 months of follow-up.

An acute tense globe tends to compress the optic nerve and can cause an irreversible loss of vision. Hence, our article aims to stress the fact that such conditions have to be managed in an emergency by performing a simple canthotomy and cantholysis to relieve the pressure on the nerve. Ultrasound-guided bleomycin is an excellent treatment of choice. However, if there is no access to B-scan or bleomycin, a wiser option would be to perform a simple decompression (canthotomy and cantholysis), as this could save an eye.

## Conclusion

Intralesional bleomycin is a very safe and noninvasive method to treat orbital lymphangioma. There are a lot of case series reporting the efficiency of this drug. Not much is reported about the efficacy of the drug with respect to the duration of the problem. However, treatment of lymphangiomas with non-invasive techniques can be tried in such tense globes irrespective of the duration.

## Notes

### Informed consent

Informed consent has been obtained from the patient’s legal guardian for the publication of this case report.

### Competing interests

The authors declare that they have no competing interests.
